# Role of survivin and its splice variants in tumorigenesis

**DOI:** 10.1038/sj.bjc.6602340

**Published:** 2004-12-21

**Authors:** F Li

**Affiliations:** 1 Department of Pharmacology and Therapeutics, Grace Cancer Drug Center, Roswell Park Cancer Institute, Elm and Carlton Streets, Buffalo, NY 14263, USA

**Keywords:** survivin, survivin-2B, survivin-ΔEx3, tumorigenesis

## Abstract

Survivin, a unique member of the inhibitor of apoptosis (IAP) protein family, is highly expressed in cancer but is undetectable in nonproliferating normal adult tissues, suggesting a potential role in tumorigenesis. Differential splicing of survivin pre-mRNA results in three new survivin variants, survivin-ΔEx3, survivin-2B, and survivin-3B. Loss of survivin-2B expression was found in the later stage of cancer development, while survivin and survivin-ΔEx3 are not, suggesting a differential role of them in tumour development. In this minireview, the author intends to summarise and discuss the current data relevant to the role of survivin and its splicing variants in tumorigenesis, which may facilitate further investigation in this interesting area.

Abnormal inhibition of physiological apoptosis during homeostasis is thought to be a critical step for carcinogenesis or tumorigenesis, although the underlying mechanism is not fully understood. In addition to the Bcl-2 protein family, which includes both antiapoptotic and proapoptotic factors, the inhibitor of apoptosis (IAP), a relative new protein family, is mainly involved in antiapoptosis. This family of proteins are characterised by a signature domain of about 70 amino acids (aa) termed baculovirus IAP repeat (BIR), which is evolutionarily conserved including virus, yeast, nematode, fly, and mammalian. Survivin, a unique member in the IAP family, is undetectable in most normal adult tissues but is highly expressed in cancer. Survivin is cell cycle regulated, and involved in both control of programmed cell death (apoptosis) and regulation of cell division. The molecular mechanism by which survivin counters against apoptosis and facilitates cell division has been extensively explored in cancer cells (Li, 2003). More effort appears to be required for the delineation of the role of survivin in tumorigenesis at the molecular level. Development of new model systems may be desired to unravel the mechanism for the function of survivin and its variants in tumorigenesis. Nevertheless, there is ample data indicating that survivin and its variants may have a differential role in tumorigenesis and cancer development. This minireview will summarise the data as a collective reference, with the intent to indicate where the current research has brought us and to help in the elucidation of future research directions in this interesting area.

## SURVIVIN IN TUMORIGENESIS

The initial indication for survivin to be involved in carcinogenesis is the observation that survivin is highly expressed in cancer as well as during development, but it is undetectable in nonproliferating adult tissues. Later studies indicated that survivin expression also occurs in very early stages of cancer, and there is a direct correlation of survivin expression with the stage of cancer and other malignant parameters. Selected studies with emphasis on tumorigenesis are discussed below.

### Role of survivin in colorectal tumorigenesis

Kawasaki *et al* (1998) reported that survivin expression reduced apoptotic index in both bcl-2-positive and -negative colorectal carcinomas, and patients with low apoptotic index in their tumours had significantly worse survival rates. This observation suggests that survivin is an independent prognostic factor. Consistently, immunohistochemical analysis of 52 colorectal carcinoma samples indicated that apoptotic index is significantly lower but PCNA labelling index is significantly higher in the 27 survivin-positive cases than in the 25 survivin-negative cases (Chen *et al*, 2004). This suggests that survivin expression not only inhibits apoptosis but it also is associated with cell proliferation.

Reports from Kawasaki *et al* (2001) and Lin *et al* (2003) further explored the role of survivin in colorectal carcinogenesis. Immunohistochemical analysis of 143 hyperplastic polyps, 171 adenomas with low dysplasia, 42 adenomas with high dysplasia, and 60 carcinomas indicated that the immunoreactivity of survivin (but not bcl-2) significantly increases in the transition from adenoma with low dysplasia to high dysplasia/carcinoma. This transition is significantly associated with a decrease of apoptotic index, and an increase of Ki-67 labelling index and microvessel density (Kawasaki *et al*, 2001). Lin *et al* (2003) showed that the positivity of survivin significantly increased in the transition from normal mucosas (0.0%) to adenomas with low-grade dysplasia (31.7%) to high-grade dysplasia (56.7%) or carcinomas (63.2%) although survivin expression was not associated with the histological differentiation grade of colorectal carcinoma. Together, these results indicate an important role of survivin in colorectal tumorigenesis, which was further supported by the study of signalling pathways relevant to colorectal tumorigenesis in other reports (see below).

Using HT-29 cell lines with inducible wild-type APC or dominant-negative TCF-4, Zhang *et al* (2001) showed that wt-APC downregulates survivin expression via suppressing *β*-catenin/TCF-4 signalling. In addition, survivin expression is preferentially in the lower crypt and inversely correlates with the expression level of wild-type APC. Consistent with the finding from Zhang *et al*, Kim *et al* (2003) reported that *β*-catenin/TCF signalling in HCT116 cells increases survivin promoter activity about 6–12-folds in comparison with that in HeLa cells lacking such signalling. Mutagenesis of the two proximal TCF-binding elements abolished survivin promoter activity by 75–79%. Survivin expression is strong in embryonic intestinal crypts, but lost in TCF-4 knockout intestinal crypts (Kim *et al*, 2003). Expression of nondestructible *β*-catenin mutants increases survivin expression and protected against UV-B-induced apoptosis (Kim *et al*, 2003). Treatment of HT-29 colon carcinoma cells with the chemopreventive drug sulindac induces *β*-catenin degradation and decreases the expression of survivin but not Bcl-2 (Zhang *et al*, 2004b).

On the basis of these findings, one mechanism for colorectal tumorigenesis appears to be that APC gene mutation results in upregulation of survivin transcription through activation of *β*-catenin/TCF-4 signalling. Survivin upregulation increases apoptosis inhibition and cell survival/overgrowth, and initiates tumorigenesis. However, in normal colorectal intestinal crypts, wild-type APC signalling suppresses survivin transcription through inactivation of *β*-catenin/TCF-4 signalling. This will increase apoptosis from crypt bottom to top, decrease cell population and block tumorigenesis. Interestingly, it was recently reported that inhibition of Wnt-2 signalling, by either a novel monoclonal antibody against human Wnt-2 ligand or Wnt-2 small interfering RNA, downregulates *β*-catenin and survivin, and induces apoptosis in Wnt-2-overexpressing human melanoma (You *et al*, 2004a) and non-small-cell lung cancer cells (You *et al*, 2004b), but not in normal cells lacking Wnt-2 expression. In addition, it was also reported that proteasome inhibitor, MG132-induced apoptosis is associated with fragmentation of *β*-catenin, reduction of TCF transcriptional activity and downregulation of survivin in hepatocellular carcinoma cells (Cervello *et al*, 2004). These findings suggest that the *β*-catenin/TCF-4/survivin pathway may not only be responsible for colorectal tumorigenesis but it may also possibly be responsible for tumorigenesis in other types of cancer.

### Role of survivin in virus infection-induced carcinogenesis

The clue for survivin involvement of virus-induced tumorigenesis comes from the observation that expression of survivin transcript was found in all the nine human T-cell leukaemia virus type I (HTLV-I)-positive T-cell lines examined and downregulation of survivin by antisense oligonucleotides inhibited growth in the HTLV-I-infected MT-1 T-cell line (Mori *et al*, 2001). Consistently, resveratrol, a chemopreventive agent commonly found in human diet, downregulates survivin expression and induces growth inhibition and apoptosis in HTLV-1-infected cell lines (Hayashibara *et al*, 2002). In addition, high survivin expression was detected in primary cells of patients with acute adult T-cell leukaemia (ATL), but not in the cells of patients with chronic ATL or normal peripheral blood mononuclear cells (Mori *et al*, 2001). It will be interesting to know whether this is associated with a difference of HTLV infection in these two types of ATL.

Immunohistochemical analysis of 73 cervical squamous tissues for survivin expression, including 31 normal, 17 low- and 15 high-grade squamous intraepithelial lesions (LSIL, HSIL), and 10 squamous cell carcinomas (SCC) from cone biopsy and hysterectomy specimens, indicated that although nuclear staining of survivin was detected in normal mucosa, LSIL, and HSIL, survivin expression is highest in human papillomavirus (HPV)-infected tissue samples. Moreover, *in situ* hybridisation of serial sections showed the colocalisation of HPV and survivin (Frost *et al*, 2002). This report indicated that the histologic correlation between nuclear staining of survivin and HPV infection suggests involvement of survivin in HPV-mediated disruption of normal cellular maturation (Frost *et al*, 2002). However, alternative roles of survivin upregulation in HPV-infected cells may exist (see below). In another report, immunohistochemical analysis for survivin expression and nested PCR plus direct sequencing for detection of HPV presence showed that survivin expression was evident in 57.1% (four out of seven) HPV^+^ (high level) and 100% (four out of four) HPV^−^ (low level) oral SCC, and 85.7% (six out of seven) HPV^+^ and 55.5% (five out of nine) HPV^−^ oral leucoplakias, and 0% (zero out of 20) control oral mucosa samples, and that survivin expression was found to be greater in HPV^+^ precancerous lesions than in HPV^−^ ones (Lo Muzio *et al*, 2004). Since HPV has been thought to be involved in the development of several oral diseases, such as premalignant mucosal lesions and oral carcinoma, these authors suggested that survivin may directly or indirectly be regulated by HPV and may be involved in HPV-induced deregulation during maturation of squamous epithelium through modulation of the apoptotic processes (Lo Muzio *et al*, 2004). Consistent with the possibility that HPV may induce survivin expression, cDNA microarray indicated that HPV E6/E7 upregulates survivin (Nees *et al*, 2000). However, Veress *et al* found the HPV E6 protein can transcriptionally upregulate survivin promoter activity over 10-folds in HeLa cells in comparison with empty vector control, while HPV E7 alone has no effect on survivin transcription (personal communication, University of Debrecen, Hungary). An interesting question is whether upregulation of survivin after virus infection is a universal phenomenon. Zhao *et al* reported that adenovirus type-2 also upregulates survivin in HeLa cells after infection (Zhao *et al*, 2003). An RNA probe derived from the adenovirus type 2-infected HeLa cells was used to examine cDNA microarrays of 12 309 unique genes. The results showed that survivin is one of the top 53 genes with significant changes (35 up and 18 down) and significantly upregulated (Zhao *et al*, 2003). Consistent with this finding, Punga and Akusjarvi (2003) showed that the adenovirus 2 E1B-55K protein upregulates survivin by binding to and blocking p53 as a transcriptional repressor on survivin promoter.

The notion that upregulation of survivin during virus infection may have a role in facilitating either virus production or virus-induced tumorigenesis comes from two additional recent reports (Marusawa *et al*, 2003; Zhu *et al*, 2003). Zhu *et al* showed that human immunodeficiency virus type 1 (HIV-1) transcriptionally upregulates survivin expression, that Vpr, an HIV-1 accessory protein, is necessary and sufficient for this effect, and that survivin may be actively involved in regulating cell viability during HIV-1 infection (Zhu *et al*, 2003). This likely facilitates virus production by increasing host cell viability. More interestingly, Marusawa *et al* showed that survivin forms complexes with a cellular protein, hepatitis B X-interacting protein (HBXIP), which was originally recognised for its association with the X protein (HBX) of hepatitis B virus (HBV). Survivin–HBXIP complexes, but neither survivin nor HBXIP individually, bind procaspase-9, preventing its recruitment to Apaf1, and thereby selectively suppressing apoptosis initiated through the mitochondria/cytochrome *c* pathway (Marusawa *et al*, 2003). Moreover, viral HBX protein also interacts with the surviving–HBXIP complex and suppresses caspase activation only in the presence of survivin (Marusawa *et al*, 2003). These observations highlighted that survivin is critical for HBV-induced apoptosis inhibition. Thus, this study provides direct evidence that survivin is likely involved in HBV pathogen-induced hepatocellular tumorigenesis.

### Additional evidence from selected reports

Lo Muzio *et al* (2003) showed that survivin is immunohistochemically present in 10 out of 30 cases (33%) of oral precancerous lesions without malignant progression, but in 15 out of 16 cases (94%) of oral precancerous lesions which evolved into full-blown SCC, and that survivin positivity appears to be an early event and a critical factor for tumor progression from precancerous lesion. Zhang *et al* showed that the immunohistochemical reactivity of survivin is 4.2% (four out of 96) in normal mammary, 5.4% (three out of 56) in cystic hyperplasia mammary, 42.7% (five out of 12) in atypical hyperplasia mammary, and 72.3% (86 out of 119) in breast carcinoma, and that the positive rates in the last two groups are statistically significant in comparison with the former two groups (Zhang *et al*, 2004a). Kim *et al* (2002) reported that in 41 cervical intraepithelial neoplasia (CIN) and SCC tissues, survivin expression is 66.7% in low-grade (CIN I), 87.5% in high-grade (CIN II and III), and 100% in SCC, which is statistically significant. These observations provide additional evidence for the involvement of survivin expression in tumorigenesis.

However, the most interesting observation comes from a study of the skin carcinogenesis using a survivin-transgenic (Tg) mouse model (Allen *et al*, 2003). Early observation from Grossman *et al* (1999)indicated that survivin is immunohistochemically expressed in 17 of 21 (81%) of basal cell carcinomas of both nodular and morpheaform subtypes, and in 24 of 26 (92%) of cutaneous SCC. Survivin is also expressed in 19 premalignant lesions of Bowen's disease and hypertrophic actinic keratosis. This suggests that survivin expression occurs early during keratinocyte transformation and may play a role in carcinogenesis. Paradoxically, Allen *et al* (2003) used DMBA carcinogen and PMA carcinogenic promoter to induce skin tumour formation in a Tg mouse model in which survivin is specifically overexpressed in skin driven by the keratin-14 gene promoter. These authors found that tumour formation was less frequent (31 *vs* 43%) and significantly delayed in Tg mice in comparison with non-Tg littermates. However, papilloma regression was not observed in Tg mice, whereas 20% of papillomas regressed in non-Tgs. One SCC was generated in Tg mice, whereas none were seen in non-Tgs. In another set of experiments, Tg mice with p53+/− background were used. Again, DMBA/PMA-induced tumour formation was less (71 *vs* 89%) and significantly delayed in Tg p53+/− animals in comparison with p53+/− non-Tgs. However, papilloma regression was also not observed in Tg p53+/− mice, whereas 10% of papillomas regressed in p53+/− non-Tgs. The rate of papilloma progression to SCC was 21% in Tg p53+/− mice in comparison with 12% in p53+/− non-Tgs (Allen *et al*, 2003). Together, these authors reported interesting results. The question is how to explain the negative effect of survivin on skin carcinogenesis and the positive effect on tumour progression. A recent report from the same research group showed that apoptosis inhibition by Tg expression of survivin enhances mutant clone creation and malignant progression during UV-B treatments in the skin carcinogenesis model, but inhibition of apoptosis by survivin in the neighbour survivin-Tg-transgenic skin cells restricts mutant clone expansion (tumour formation) (Zhang *et al*, 2004c). This may provide an explanation for the paradoxical results above. Anyway, the model will be significantly strengthened, if it could gain an insight into a molecular basis to explain why apoptosis in the neighbour normal cells is so important for the mutant clone to be expanded (tumour growth) in skin.

Alternatively, in my personal view, the results from Allen *et al* may also suggest that artificial overexpression of survivin in normal tissues may show a different role from survivin expression in abnormal precancerous and cancerous tissues. For example, the artificial overexpression of survivin in normal mouse skin may counter against the process of DMBA/PMA-induced carcinogenesis. However, through a selection process driven by DMBA/PMA treatments, individual skin cells with additional genetic and/or biochemical abnormalities will give survivin a different background for function. In the latter environment, survivin plays a positive role in tumour growth/progression for promoting papilloma regression and promoting conversion to SCC.

## SURVIVIN VARIANTS IN TUMORIGENESIS

Human survivin gene has four dominant (1, 2, 3, and 4) and two hidden (2B and 3B) exons. Alternative splicing of its pre-mRNA produces four different mRNAs, which encode four distinct proteins, survivin, survivin-2B, survivin-ΔEx3 (Mahotka *et al*, 1999), and survivin-3B (Badran *et al*, 2004). Survivin (142 aa) is derived from exons 1–4; survivin-2B (167 aa) has an additional 23 aa derived from a 69-bp cryptic exon (2B) within intron 2, which is spliced into survivin mRNA in frame between exons 2 and 3; survivin-ΔEx3 (137 aa) is derived from exons 1, 2, and 4, a frameshift read-through variant due to exon 3 escape; and the recently reported survivin-3B (120 aa) consists of the N-terminal 113 aa of survivin (coded by exons 1, 2, and 3) plus seven new aa sequences at C-terminal tail encoded by a DNA sequence (exon 3B) from intron 3 of survivin. Currently, there is no strong evidence suggesting that survivin splicing variants are involved in tumorigenesis. However, Islam *et al* (2000) reported that the expression of survivin-2B is predominant in some neuroblastoma with a good prognosis, but it is expressed at low levels in most malignant tissues. In renal cell carcinomas (RCC), while the expression levels of survivin and survivin-ΔEx3 did not show a decrease in late tumour stages in comparison with the early and intermediate tumour stages in 57 clinical RCC samples, survivin-2B expression was significantly decreased in late tumour stages (Mahotka *et al*, 2002a), indicating a possible unfavourable role of survivin-2B in cancer development. In gastric cancer, the expression of survivin-ΔEx3, survivin-2B, and survivin (dominant transcript) was found in cancer tissues, irrespective of their histological type, grade, or stage (Krieg *et al*, 2002) (Meng *et al*, 2004). However, survivin-2B expression was significantly decreased in later tumour stage (III+IV) in comparison with early stage (I+II) (Krieg *et al*, 2002; Meng *et al*, 2004), and inversely correlated with tumour differentiation and invasion (Meng *et al*, 2004). Interestingly, although Krieg *et al* (2002) reported that the expression of survivin and survivin-ΔEx3 remained unchanged in different stages of cancer, Meng *et al* (2004) showed that the expression level of survivin-ΔEx3 is inversely correlated with apoptotic index. It was also reported that survivin-2B expression was dominant in benign brain tumours in comparison with the malignant ones (Yamada *et al*, 2003), and that survivin-ΔEx3 expression was prominent in comparison with survivin-2B expression in survivin-expressing acute lymphocytic leukaemia (ALL) and chronic lymphocytic leukaemia (CLL) patient bone marrow samples (Nakagawa *et al*, 2004). Structurally, a novel antiapoptotic protein encoded by open reading frame K7 of Kaposi's sarcoma-associated herpesvirus bears a resemblance to survivin-ΔEx3 (Wang *et al*, 2002), further suggesting a possible antiapoptotic role for survivin-ΔEx3. In addition, the different subcellular localisation of survivin-ΔEx3 (in nucleus) and survivin-2B (in cytoplasm) (Mahotka *et al*, 2002b) also suggests their potential different roles. Collectively, these observations indicated that survivin-ΔEx3 and survivin-2B may play an opposing role in tumour progression and/or tumorigenesis.

Interestingly, in soft tissue sarcoma (STS), 36 of 56 STS (64%) were survivin transcript-positive, and 15 of the 36 survivin-positive samples were survivin-ΔEx3 transcript-positive, but survivin-2B transcripts were undetectable (Kappler *et al*, 2001). Similarly, among the 79 gastric tumour samples, the expression of survivin, survivin-2B, and survivin-ΔEx3 was 79 (100%), 62 (78.5%), and 51 (64.6%), respectively (Meng *et al*, 2004). Additionally, studies from Islam *et al* (2000) indicated that while the expression of survivin was downregulated during retinoic acid-induced apoptosis in CHP134 neuroblastoma cells, survivin-2B expression was unchanged or slightly increased during apoptosis. These observations suggest that the expression of these survivin variants could be differentially regulated. Consistent with this notion, recent studies from Zhu *et al* (2004) showed that one of the mechanisms for p53 to induce apoptosis in ALL cells is through differential modulation of survivin and its variants. Doxorubicin-activated p53 upregulated survivin-2B but downregulated survivin and survivin-ΔEx3. Therefore, from a cancer prevention and therapeutics point of view, the idea that survivin-2B may act as a natural antagonist against the function of survivin and/or survivin-ΔEx3 may lead to novel approaches for cancer prevention and/or therapeutics through differential modulation of the expression of survivin and/or its variants.

## SUMMARY REMARKS

The potential role of survivin in colorectal tumorigenesis appears to be well defined. Many observations also indicate a role of survivin in virus infection-induced carcinogenesis. In addition, studies of normal and different stages of precancerous and cancerous tissues in many other types of cancer also suggest a role of survivin in tumorigenesis. However, the study of DMBA/PMA-induced carcinogenesis in a mouse skin model (with or without a p53+/− background) that artificially overexpresses survivin in skin using a skin-specific keratin-14 gene promoter, paradoxically revealed that tumour formation was less frequent and significantly delayed in survivin-Tg mice in comparison with non-Tg littermates, although Tg mice promote papilloma progression into SCC after tumours are initiated from DMBA/PMA treatment. This interesting observation could be due to the apoptosis inhibition by survivin in the neighbour normal cells, which prevents mutant cell expansion (tumour growth), but it may also suggest that the potential role of survivin in normal skin is different from the role in precancerous and cancerous tissues. Therefore, studies of the different role and functional mechanism of survivin between normal and cancerous tissues will help to answer the remaining questions. New research approaches and/or model systems will be required to elucidate the distinct mechanisms by which survivin functions and is regulated in cancer *vs* normal cells, which will be essential for development of novel and highly effective approaches for cancer prevention and therapeutics. In addition, although data from several reports suggest a potential role of survivin-2B in countering against the function of survivin and/or survivin-ΔEx3 in tumour developments, much more needs to be done before deriving a definitive conclusion.
